# Functional Heatmap: an automated and interactive pattern recognition tool to integrate time with multi-omics assays

**DOI:** 10.1186/s12859-019-2657-0

**Published:** 2019-02-15

**Authors:** Joshua R. Williams, Ruoting Yang, John L. Clifford, Daniel Watson, Ross Campbell, Derese Getnet, Raina Kumar, Rasha Hammamieh, Marti Jett

**Affiliations:** 1 Advanced Biomedical Computational Science, Frederick National Laboratory for Cancer Research sponsored by the National Cancer Institute, Frederick, MD 21702-5010 USA; 2 Integrative Systems Biology Program, US Army Center for Environmental Health Research, Fort Detrick, Frederick, MD 21702-5010 USA

## Abstract

**Background:**

Life science research is moving quickly towards large-scale experimental designs that are comprised of multiple tissues, time points, and samples. Omic time-series experiments offer answers to three big questions: what collective patterns do most analytes follow, which analytes follow an identical pattern or synchronize across multiple cohorts, and how do biological functions evolve over time. Existing tools fall short of robustly answering and visualizing all three questions in a unified interface.

**Results:**

Functional Heatmap offers time-series data visualization through a Master Panel page, and Combined page to answer each of the three time-series questions. It dissects the complex multi-omics time-series readouts into patterned clusters with associated biological functions. It allows users to identify a cascade of functional changes over a time variable. Inversely, Functional Heatmap can compare a pattern with specific biology respond to multiple experimental conditions. All analyses are interactive, searchable, and exportable in a form of heatmap, line-chart, or text, and the results are easy to share, maintain, and reproduce on the web platform.

**Conclusions:**

Functional Heatmap is an automated and interactive tool that enables pattern recognition in time-series multi-omics assays. It significantly reduces the manual labour of pattern discovery and comparison by transferring statistical models into visual clues. The new pattern recognition feature will help researchers identify hidden trends driven by functional changes using multi-tissues/conditions on a time-series fashion from omic assays.

**Electronic supplementary material:**

The online version of this article (10.1186/s12859-019-2657-0) contains supplementary material, which is available to authorized users.

## Background

Many diagnostic and therapeutic studies are rapidly adopting a time-series experimental design including microarray gene expression and RNA-seq. The number of time-series transcriptome data sets have grown exponentially over the last decade, enabling researchers to identify the complete set of activated genes in a biological process, to infer rates of change or causal effects, and to model dynamic events in the cell [1]. Researchers are particularly interested in transcriptomic patterns that correlate with clinical or experimental observations. However, the traditional hierarchical clustering heatmap [2], k-means clustering [3], or biclustering [4] do not consider time dependent patterns innately, and thus are inadequate to search specific patterns that underpin mechanisms of biology. Few common statistical models are currently used to fit time series data on other observations. These tools include autoregressive models [5, 6], Bayesian approaches [7], self-organizing maps [8], and triclustering [9]. All of these models result in global parent clusters of components, while many distinct subpatterns may be neglected or over fitted due to assumptions and inherent biases built in the statistical models of choice. For example, lower degree polynomial autoregressive models tend to have only few patterns while higher degree polynomial modes can lead to over fitting in short time-series. Phang et al. proposed a trajectory clustering method that defined gene profiles by the direction of change between adjacent time points, and concatenated the direction into a key [10]. This trajectory method is an example of the symbolic representation method that has been popularly used in video streaming. The symbolic representation discretizes the profile and maps it to symbols, thus gene profiles can be represented as a concatenation of symbols. The discrete representation becomes very powerful in matching and comparing patterns. For example, we have sectional gene expression data, and the genes may be discretized into three levels of Fold Change (FC) between treatments and controls: “+” if FC ≥ 2; “-” if FC ≤ − 2; and “0” if − 2 < FC < 2. However, one can also design more levels or designate the slope of adjacent time points as symbols, and use different cutoffs for levels. Most researchers compute differentially expressed genes (DEGs) in terms of the t-test *p*-value at individual time points and compare the common DEGs across time. This is also an example of symbolic representation, such as up−/down-regulated DEGs that are “+” and “-”, respectively, and the rest are “0”. When all these characters are concatenated into a string, such as ‘++−’, then the string means a temporal profile ‘up’ ‘up’ ‘down’. We then group the genes by their profile and display in a heatmap. This heatmap can help researchers answer, but are not limited to, the following questions: 1) the collective trends (the patterns that most genes follow), 2) the consistent trends (the genes that exhibit identical patterns across multiple datasets), 3) the sequential trends (the cascade response of genes across time or across conditions) and 4) the stage trends (early-responsive or late-responsive genes). Answering these questions in multi-tissue and multi-condition time-series data becomes a multi-dimensional comparison problem (e.g., N-dimension Venn diagram) and it is difficult to trace genes with the same pattern of expression in current tools. In this paper, we developed a comprehensive interactive transcriptomics analysis and visualization tool, Functional Heatmap, based on the concept of symbolic representation. Functional Heatmap offers time-series data visualization through a Master Panel page and a Combined page to answer each of the multi-dimensional time-series questions. All analyses are interactive, searchable, and exportable in the form of heatmap, line-chart, and text, and the results are easy to share, maintain, and reproducible on the web platform. The pathway enrichment can also be conducted based on a merged pathway database that collapses highly similar pathways curated from different resources, including KEGG version 80 [11], Wiki pathway [12], Biocarta [13], Reactome [14], and GSEA [15]. To avoid the potential bias of super large pathways such as cancer pathway and duplicate pathways curated from different resources, we trimmed and merged the pathway database before further pathway enrichment. First, we filtered out the super large pathways with thousands of genes. Next, we calculated the overlap rate (Eq. 1) between each pathway pair *i* and *j*,

1 $$ Overlap\left(i,j\right)=\min \left({Length}_i,{Length}_j\right)/\left({Length}_i+{Length}_j-{Length}_{\left(i\cap j\right)}\right) $$

Then the overlap rates were used as the distance matrix in hierarchical clustering with average linkage. All the tree under height 1.5 (roughly corresponding to 85% overlap rate) were merged into new pathways. The pathway enrichment was conducted by standard one-side hypergeometric test.

## Implementation

**Fig. 1:**
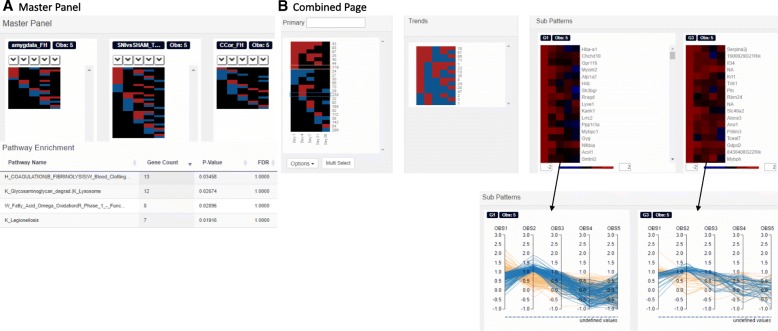
Available display modes in Functional Heatmap. **a** Master panel page displays side-by-side visualizations of several heatmaps simultaneously. A given row can be selected to display pathway enrichment. **b** Combined page displays the primary heatmap of all the patterns combined on the left, with trends in the middle and the subpatterns of gene expression to the side. Below are the flipped subpatterns to display line charts of the data

Functional Heatmap is hosted online at https://bioinfo-abcc.ncifcrf.gov/Heatmap/. It is written in PhP 5 and open-source JavaScript libraries D3.js and jquery.js. Since the Functional Heatmap software application is completely web-based, there are no installation requirements and no restrictions on operating systems. The software can be launched on any computer system that is connected to the internet and capable of running one of the current web browser applications with JavaScript capabilities enabled (i.e., Internet Explorer, Google Chrome, Mozilla Firefox, Safari). Mozilla Firefox or Google Chrome are recommended for use with the tool. Functional Heatmap efficiently incorporates robust clustering of genes based on expression profiles, heatmap visualizations, and annotation of like-groups together in one web-based tool as compared to other tools (Table 1). Functional Heatmap supports abstraction of data multi-dimensionality by representing observations (e.g., individuals or time points) as a primary heatmap, and displaying relative correlations with a feature of interest. Each panel in the primary heatmap encapsulates a subpattern of the individual gene expression values unique to that data point (Fig. 1a).

**Table 1:**
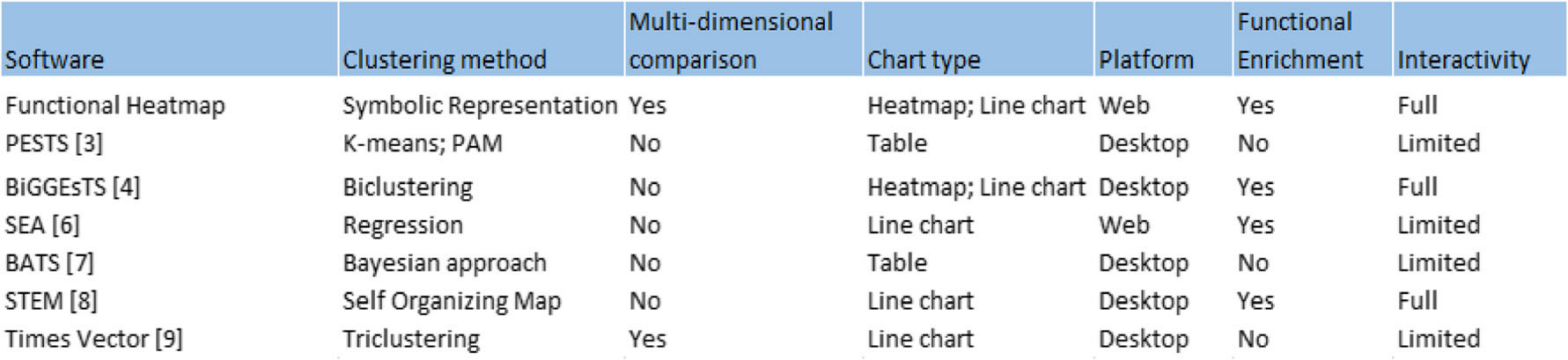
Comparison to existing tools

The users must provide an input file that contains ID, Entrez Gene (optional), Symbol (optional), *P*-value (optional), and fold change (FC) for each time point (see Additional file 1: Supporting Material User Manual, Additional file 2: Sample input file S2). The users can select different significance cutoffs in the filter menu to down-select genes for the clustering analysis. The users also can apply other DEG analysis tools, such as EDGE [16], and upload the DEG list the Functional Heatmap. Users may notice that there are many miscellaneous applications for Functional Heatmap besides genetics. These include multi-dimensional continuous time-series data from biological analytes (protein, metabolite, microbiome, etc.), financial data, or engineering data.

### Availability and requirements

Functional Heatmap is publicly available at https://bioinfo-abcc.ncifcrf.gov/Heatmap/. An illustrative video for Functional Heatmap is available in Additional file 3.


**Additional file 3:** Illustrative video. (MP4 12800 kb)


Operating systems: Windows/OSX

Programming language: PHP and JavaScript

Browsers: IE 9, Firefox 31, Chrome 31, Safari 5.1, Opera 24, Opera Mini 8, iOS safari 7.1, Android Browser 4.4, or later.

## Results

Functional Heatmap offers two pages: 1) Master Panel page, and 2) Combined page. The Master Panel page (Fig. 1a) displays the patterns from each file uploaded side by side. The Combined page (Fig. 1b.) combines the contents of each file in the Master Panel and displays genes that follow the same pattern across cohorts. These clusters of genes behave the same and are synchronized independent of the conditions being evaluated. Patterns of association with a measured statistic (such as disease severity) can be visualized in the primary heatmap (Fig. 1b, far left panel), while the corresponding gene expression patterns can be simultaneously viewed on the Subpatterns heatmaps (Fig. 1b, far right 2 panels). Additionally, each pattern in the primary heatmap can be further broken down into trends and the heatmap trends for that pattern are displayed between the primary and subpattern heatmaps (Fig. 1b). The trends show the expression difference across time points. If there is a gene with fold changes 2, 3 and 4 at time points 1, 2, and 3, respectively, this would have an upward trend because the values are increasing. Conversely if there was a gene with fold changes 5, 4, 3 at time points 1, 2, and 3, respectively, this would have a downward trend. Both of these genes would be in the primary pattern of “up up up” or “+++” symbol, which is why this further breakdown is necessary to distinguish between the complex behavior of genes-of-interest in a more precise manner. By selecting a particular trend, such as the downward trend, the genes in the subpattern with a matching trend will be displayed. This allows the user to view the groups of trends that genes follow based on a particular higher level parent pattern and can filter out all other trends to see exactly which genes of the primary pattern follow a particular trend-of-interest. As illustrated in the example, such a capability allows the user to see particular sets of genes that may have had a spike in expression early on but were on a steady decline or back to a normal state after a given time point. The user can also toggle the subgroup heatmaps (Fig. 1b) to show data in the form of a line chart of expression levels. The rest of the genes from the primary heatmap will still be visible as faded lines, when a trend is selected. A searchable list of genes comprised of each level of the heatmap is dynamically displayed when the user selects a pattern in the primary heatmap.

**Fig. 2:**
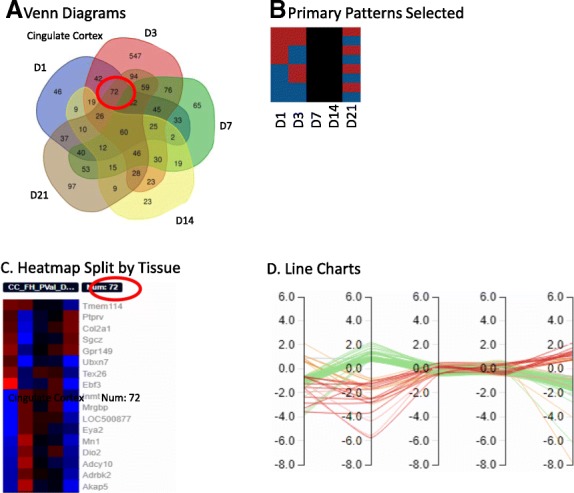
Viewing Overlap. **a** Traditional Venn diagrams showing the overlap between genes across time for two different tissues. The circled overlap is what is displayed in sections **c** and D.B) Primary patterns selected which have expression +/− on columns 1, 2 and 5. **c** Shows the gene expression heatmaps split out by tissue. **d** Line charts for the heatmaps above where each column is day 1, 3, 7, 14 and 21, respectively. The y-axis is log base 2-fold change values. Line colors represent the corresponding row selected in **b**

To further illustrate the capabilities of the Functional Heatmap as compared to traditional Venn diagrams, we present data from a study in rats which evaluated gene expression differences in the cingulate cortex across days 1, 3, 7, 14 and 21, post-injury in a chronic pain model. Here, one can use the traditional Venn diagram to show the overlap in DEG identities at the different time points in this tissue (Fig. 2a). However, the Venn diagram is neither able to stratify those genes into different expression patterns, nor can the identities of the genes be readily displayed. Using Functional Heatmap’s Combined page, we can see that the 72 DEGs common on days 1, 3, and 21 within the cingulate cortex can be further stratified into eight different combinations of up/down-regulation across the three selected time points (Fig. 2b). While a Venn diagram only shows the total number of DEGs in that group (Fig. 2a), Functional Heatmap allows the user to discern trends within those 72 genes that may signify underlying biological functions. This function enables the user to dynamically select the type of overlap of interest such as genes that overlap across time, but are highly upregulated, and then return that particular subset of genes and pattern information to the user. The user can further see the identity and expression pattern of these overlapping genes (Fig. 2c), as well as the corresponding line chart (Fig. 2d), by selecting the flip heatmap option.

In addition, automatic pathway enrichment information for sets of genes is generated at each level of analysis, allowing users to efficiently interpret the selected patterns and view the biological processes underlying the data in greater detail and more quickly than with any previous tools. Data can be sorted in a variety of ways quickly and intuitively revealing patterns that would otherwise remain undetected using traditional static visualization tools. In additional, Functional Heatmap can consolidate differing numbers of data points with their mean. For example, suppose an experiment compares multiple mouse strains with differing numbers of time points for each mouse. Functional Heatmap can consolidate the time points by taking the mean values at the two time points seamlessly within the analysis, removing the need for extensive data preprocessing. Once each experiment has an identical number of time points, they can easily be compared.

Functional Heatmap application provides users with a robust automated, yet interactive, analytical framework that requires no prior computational expertise. Researchers and bioinformaticians alike can easily access a combination of powerful computational tools without having to develop a customized code to handle each use case. By intuitively answering the three most widely sought after questions from time-series experiments, Functional Heatmap allows scientists to rapidly and reproducibly extract biological meaning and create publication-quality figures from their time-series data simultaneously by using a single tool. Functional Heatmap represents a one-stop shop for analyzing high-throughput gene expression experiments. Furthermore, by encapsulating all the computational elements of the tool on a remote server, Functional Heatmap is universally compatible, and offers high-resolution and comprehensive gene expression analysis resources to any scientist with an internet connection regardless of their local resource availability. Finally, by alleviating the need for the user to write and maintain customized analysis scripts, Functional Heatmap presents a greatly simplified platform for reproducing large-scale data analyses. A detailed comparison to available time-series tools is listed in Table 1.

In the future, Functional Heatmap will connect to the time-series network suite PanoromiX (https://bioinfo-abcc.ncifcrf.gov/panormics/), which allows the users to review dynamic changes of different functional modules in the progression of biological conditions. Furthermore, more statistical comparison and pattern recognition tools will be implemented to the back-end server.

### Example from an ongoing multidimensional study

**Fig. 3:**
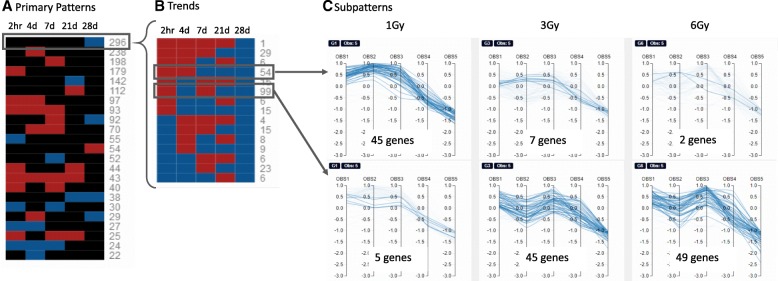
Combined Page with Example. **a** The primary heatmap of all the patterns sorted by number of genes per pattern, highest to lowest. **b** The trends which come from the selected pattern in the Primary Patterns heatmap. The trends make up the 296 genes in the selected pattern. **c** The subpatterns filtered by the 99 and 54 genes from the trends. This allows the user to visualize which subpatterns of the 54 and 99 genes are associated with. This figure shows that most of the 54 genes showing a spike up then a drop are mostly from the 1Gy dose. The most abundant trend of 99 genes are mostly from the high 6Gy dose followed closely by the 3Gy dose

The following provides an actual example of the use of Functional Heatmap to facilitate analysis of a multidimensional transcriptomic dataset. Recently, investigators at our institution, along with collaborators, have conducted a radiation dose response (1, 3, and 6 Gy [Gy] X-ray exposure) and time course (2 h, and 4, 7, 21, and 28 days post-exposure) experiment in mice, in an effort to gain detailed insight into the effects of ionizing radiation (IR) on skin. A comprehensive assessment of the transcriptome of the skin was conducted across all doses and time points, using DNA microarrays [manuscript under review]. The differentially expressed genes (DEGs) were identified as log fold mRNA expression values for each dose and time point, comparing irradiated to time-matched non-irradiated controls. The DEG lists (FC > 2, *P* > 0.05) for each dose were used to generate the Master Panel of expression patterns, and then combined to generate the primary heatmap of all patterns (Fig. 3a, depiction of Combined Page). The primary patterns were sorted by descending DEG number (Sort by count), and the most abundant pattern, containing 296 genes, was chosen for identification of trends (Fig. 3b). Genes fitting this pattern have a differential expression of less than 2-fold between irradiated and non-irradiated controls at every time point (black color) except for the last time point at day 28 (blue color), where expression was twofold or less in the irradiated group compared to controls. The first and second most abundant trends, containing 99 and 54 DEGs, respectively, were next chosen for assessment of subpatterns for each dose (Fig. 3c). Interestingly, the 99 DEGs having the trend of + − + − -, were predominantly contained in the 3Gy and 6Gy treated skin groups, with only five genes matching this trend for the 1Gy treated skin. Conversely, the 54 DEGs having the trend of ++ − --, were predominantly present in the 1Gy treated skin (45 of the 54 DEGs). This comparison reveals a striking difference in expression trend between the 1Gy dose and the others. Further analysis of these specific DEGs, as well as others that are being identified using the Functional Heatmap, is ongoing. It is anticipated that this tool will both focus the effort and speed the discovery of the underlying biology and the corresponding gene networks that are most important for understanding the effects of varying doses of IR on skin over time.

## Conclusions

Functional Heatmap is an automated and interactive tool to enhance pattern recognition on time-series multi-omics assays. It reduces the manual labour of pattern discovery and comparison by transferring statistical models into visual clues. The new pattern recognition will greatly help the researchers identify hidden trends of functional changes using multi-tissues/condition time-series omic assays. Researchers can easily access a combination of powerful computational tools without having to develop customized code to handle each use case.

## Additional files

Additional file 1: User Guide. (DOCX 3051 kb)

Additional file 2: A sample input file Additional file. (TXT 42 kb)
