# Supplementary material for: Functional Heatmap: an automated and interactive pattern recognition tool to integrate time with multi-omics assays
Source: BMC Bioinformatics. 2019 Feb 15;20:81. doi: 10.1186/s12859-019-2657-0 (PMC6377781; doi:10.1186/s12859-019-2657-0)
Supplement: Supplementary file 2 — User Guide. (DOCX 3051 kb) [file 12859_2019_2657_MOESM1_ESM.docx]

**USER’S**

**MANUAL**

*Functional Heatmap*

**U.S. Army Center for Environmental and Health Research**

August 29, 2018

**Revision Sheet**

| **Release No.** | **Date** | **Revision Description** |
| --- | --- | --- |
| Rev. 1 | 5/22/17 | User’s Manual Version 1 |
| Rev. 2 | 9/1/17 | User’s Manual Version 2 with Pathway, Filter, Line Chart added |
| Rev. 2.1 | 9/19/17 | Added data validation features |
| Rev. 3.1 | 8/29/18 | Added new trend images, download and changed verbiage |
|  |  |  |
|  |  |  |
|  |  |  |

**USER'S MANUAL**

**TABLE OF CONTENTS**

Page #

1.0 GENERAL INFORMATION

1.1 System Overview

1.2 Authorized Use Permission

1.3 Points of Contact

1.3.1 Information

1.4 Organization of the Manual

1.5 Acronyms and Abbreviations

2.0 SYSTEM SUMMARY

2.1 System Configuration

2.2 Data Flows

2.2.1 Data Flow Single File 2-1

2.2.2 Data Flow Multiple Files 2-1

3.0 GETTING STARTED

3.1 System Menu

3.1.1 Features

3.1.2 Examples

3.1.3 Get Started

3.1.4 Contact

4.0 USING THE SYSTEM (ONLINE)

4.1 Features 4-1

4.2 Examples 4-1

4.2.1 Bone Study Example ..2

4.2.2 Data File Template Example Section 1 4-3

4.2.3 Data File Template Example Section 2 4-3

4.3 Get Started 4-3

4.3.1 Upload Data 4-3

4.3.2 Data Validation 4-4

4.3.3 Comparison Page 4-5

4.3.4 Combined Page 4-5..7

4.4 Contact Page 4-7

**1.0 GENERAL INFORMATION**

# GENERAL INFORMATION

## 1.1 System Overview

Functional Heatmap: A comprehensive automated transcriptomics analysis and visualization tool:

1. Automating pattern recognition
2. Generate fold change curve
3. Consider within sample variance
4. Functional Enrichment
5. Search genes
6. Pathway Enrichment Views
7. Multiple dataset comparison analysis
8. Interactive Parent and Child heatmaps
9. Traditional and Custom pattern sorting
10. Abstract data multidimensionality through heatmap
11. Single file or multi file upload
12. Highlighting genes associated with pathways

## 1.3 Authorized Use Permission

This application is a web based application and is open for anyone to use. Currently everyone is authorized to use the system, and the application does not require logging into a system.

## 1.4 Points of Contact

### 1.4.1 Information

For questions, comments or concerns with the software please contact:

Joshua Williams at [Joshua.Williams2@NIH.gov](mailto:Joshua.Williams2@NIH.gov)

Visualization Development & UX/UI Design

Daniel Watson at [Daniel.Watson@NIH.gov](mailto:Daniel.Watson@NIH.gov)

Visualization Development & UX/UI Design

Ruoting Yang at [Ruoting.Yang@NIH.gov](mailto:Ruoting.Yang@NIH.gov)

Algorithm & Visualization Development

## 1.5 Organization of the Manual

Provides a list of the major sections of the User’s Manual (1.0, 2.0, 3.0, etc.) and a brief description of what is contained in each section.

## 1.6 Acronyms and Abbreviations

List of the acronyms and abbreviations used in this document and their meaning.

VPN – Virtual Private Network

NIH – National Institute of Health

**2.0 SYSTEM SUMMARY**

# SYSTEM SUMMARY

## 2.1 System Configuration

The system is being hosted on the NIH servers and the app requires access to the NIH server where the database is being stored. The user can access the site from anywhere they have internet access without the requiring the use of a VPN. This system receives as input the users’ data in the form of text files and returns a visualization of their data in the form of a heatmap.

## 2.2 Data Flows

The data for the application flows in one of two ways in the beginning during the pre-processing step and then comes together afterwards. Each process is described in more detail later in section 3.0.

### 2.2.1 Data Flow Single File

The user will upload a data file (or multiple data files) which already have all their observation data calculated and just need patterns added to their files. The file can optionally have p-values for each observation point. This Functional Heatmap automatically takes each observation point for each of the probes and assigns an up (+), down (-) or unchanged (0) symbol for that data point based on the threshold. Once all the data points for a particular probe/row are assigned, then the collection of these symbols are concatenated together to create the pattern for that row, which is then uploaded to a database for later usage.

### 2.2.2 Data Flow Multiple Files

The user uploads at least three files; annotation, information and data files. Using the information file, the application can read the data file and make the necessary fold change calculations to create the patterns as either up (+), down (-) or neutral (0) for each data point, and then concatenate them together for each probe to determine its pattern, which is then uploaded to the database for later usage. The annotation file is used to map the gene ID and gene names to the probes in the data file.

**3.0 GETTING STARTED**

# GETTING STARTED

## 3.1 System Menu

When the user first goes to the Home screen of the web application they will see four options available to them which are Features, Examples, Get Started, and Contact. Each link will take you to the appropriate page, for example Features will take you to the page with our Features. The Examples page has a link to an example and some example files you can download. The Get Started link is where you will get started with uploading your data, and lastly, the Contact page has all of our contact information.

### 3.1.1 Features

This page lists all the features that the software provides which include but are not limited to:

• Online web application tool.

• Heatmap capable with search feature.

• Comprehensive transcriptomics analysis.

• Incorporated clustering and pattern assignment.

• Gene Ontology (GO) identification.

• Pathway Enrichment table views.

### 3.1.2 Examples

On the examples page there is a link to the Bone Study Example. Following this link will take you to the combined page where you can view the heatmap made for this particular data set. This allows you to see what the system is capable of and view patterns and gene heatmaps while seeing the pathway information, which is connected to those particular genes.

There is also a Data File Templates section which outlines the two types of data that the application currently accepts as input and provides examples that you can download and look at for reference.

### 3.1.3 Get Started

On the Get Started page you have two options to choose from. Either your data just needs to be analyzed and have patterns added, or your data needs to have calculations performed and then patterns added. You can also download an example file which shows what your data needs to look like.

### 3.1.4 Contact

This page provides you with the names and email addresses of team members who can help if you have any problems, or questions/comments.

**4.0 USING THE SYSTEM (ONLINE)**

# USING the SYSTEM (ONline)

This section provides a description on using the online web application and the different available options.

## 4.1 Features

The Features page simply outlines all the features that this web application currently has available.

## 4.2 Examples

The Examples page is broken down into two sections, the first is the examples section which has a bone study example you can click on and view the page with this example loaded for you (Fig 4.2.A).


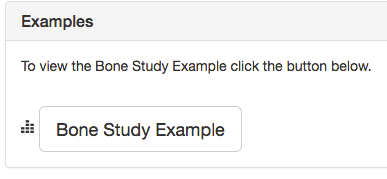


Fig. 4.2.A – Examples section where the user can select to view the Bone Study Example.

The second section of the examples page is the Data File Templates, which itself is subdivided into two smaller sections, depending on the format of your data. Refer to section 4.2.2 and 4.2.3 for more details.

### 4.2.1 Bone Study Example

This bone study example allows you to view the patterns that came from this bone study data set. You can select different patterns which will generate the gene heatmaps on the right. As you select the patterns the application takes the genes associated with the selected pattern and then performs pathway enrichment analysis on the genes and returns a list of pathway names associated with that list of genes (Fig 4.2.B).


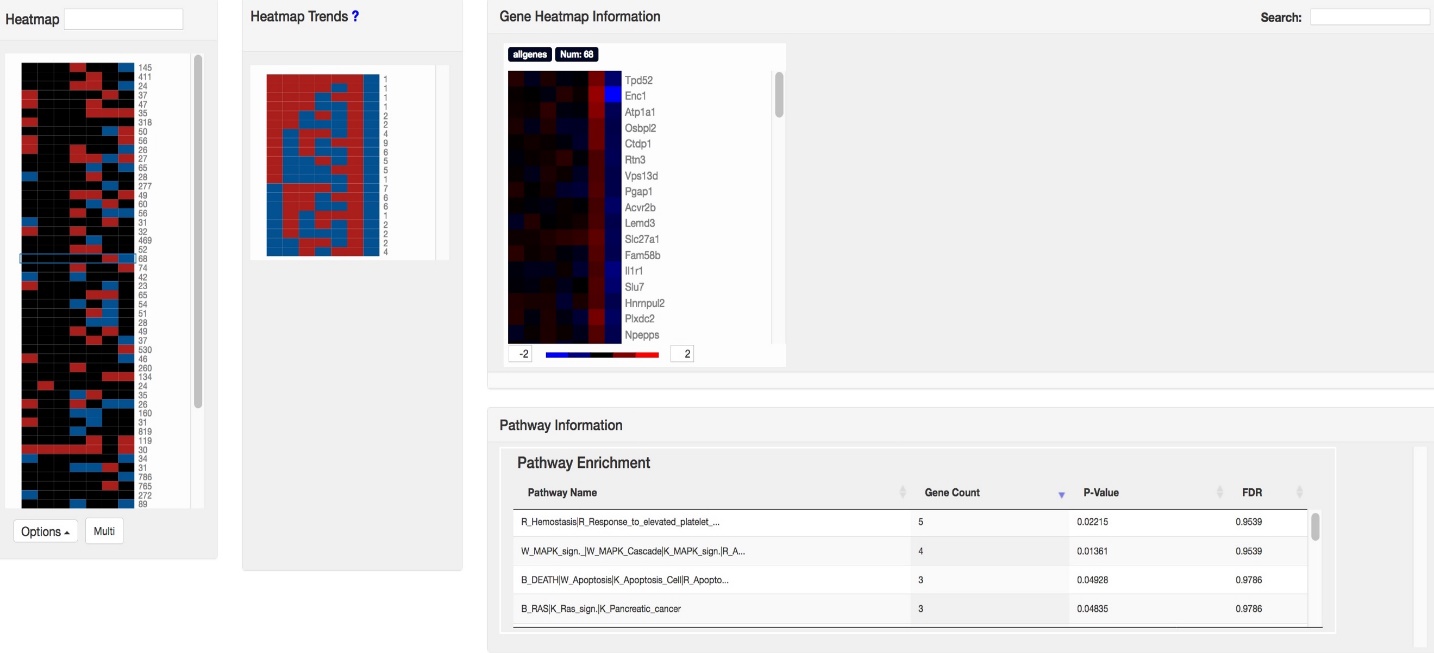


Fig. 4.2.B – Combined page showing the primary heatmap (left), trend patterns (middle), the gene heatmap (right), and the pathway table (bottom).

You can select a pathway of interest in the “Pathway Enrichment” table and the application will then highlight all the genes which are associated with that pathway and make them the only ones visible in the gene heatmap (Fig 4.2.C).


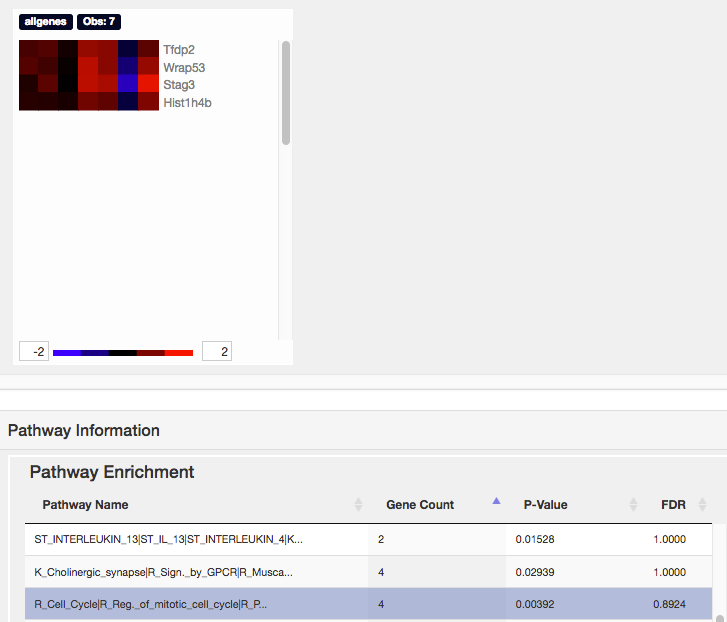


Fig. 4.2.C – Pathway selected in table reveals the genes associated with that pathway and hides all other genes.

### 4.2.2 Data File Template Example – Section 1

The first section in the data file templates section is for the users who just need patterns added to their data. The application shows what your data file(s) header must include for the application to know where your data is located. Your data must also be in comma delimited text file format for the application to work. You can download an example file (Fig 4.2.D) to see how the header needs to be setup and the way your data needs to be formatted.


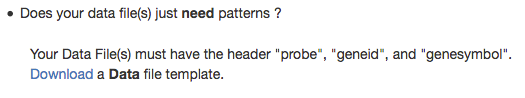


Fig. 4.2.D – First section of the data file templates where you can download an example template file.

### 4.2.3 Data File Template Example – Section 2

The second section in the data file templates section is for the users who have their data broken up into data file(s), annotation, and information files and need patterns added to their data along with fold change analysis. This shows what the three headers need to be for each of the files. The data file(s) needs to have the header “probe”, as well as match all the sample names from the information file. This allows the application to map the sample names to the data file. You can download an example of all the files and their structure on this page (Fig 4.2.E). The annotation file needs to have at least all the probe names that are in your data files.


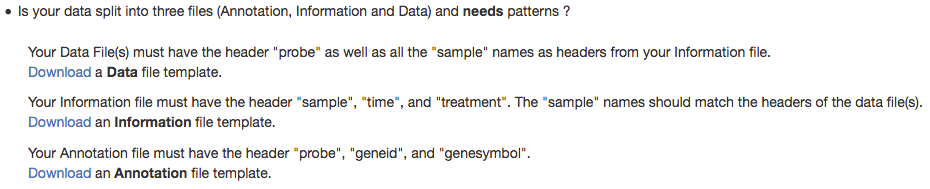


Fig. 4.2.D – Second section which shows header requirements for the three files as well as examples you can download of each file.

## 4.3 Get Started

This is the main part of the web application because this is where you will be uploading your data to the application. The application currently supports two data formats, the first is where your data simply needs patterns added and has already been preprocessed. This means you have probes, gene IDs, gene symbols, and your observation data and just need the observation data analyzed and have the application automatically apply patterns to it. The second format the application supports is when you have your data broken up into annotation, information and data file(s). Refer to section 4.2.2 and 4.2.3, respectively, for examples of these two data file formats and how they need to be setup.

### 4.3.1 Upload Data

Once you are ready to upload your data file(s) select which option best matches your data file format. You either have data file(s) which need patterns added to them (Fig 4.3.A) or you have data broken up into three or more files and need to upload them to their correct area (Fig 4.3.B).


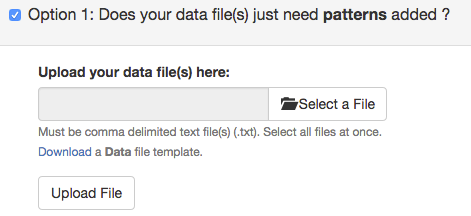


Fig. 4.3.A – First section of the data file templates where you can download an example template file.

Once you select the option that best matches your data, you upload your comma delimited text files to the appropriate area. Click “Upload File(s)” button at the bottom and wait while your data is uploaded and processed. The amount of data in your files will determine how long it takes to upload your data and process it. Once your data has finished uploading and processing, you will be redirected to the data file validation page, which is covered in section 4.3.2.


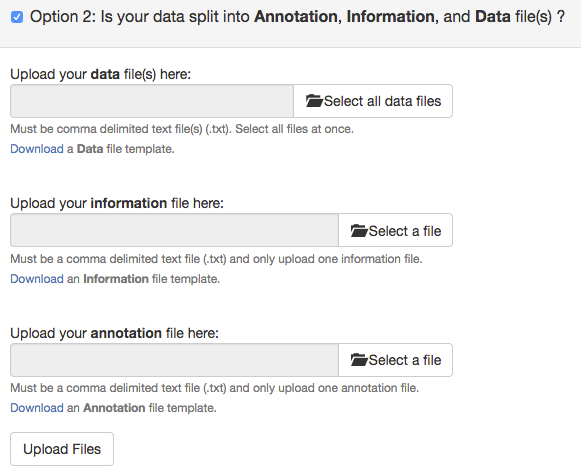


Fig. 4.3.B – First section of the data file templates where you can download an example template file

### 4.3.2 Data Validation

The data validation page is where you are directed after your data has finished uploading. This shows the number of observations found within each file (Fig 4.3.C). If there were any errors encountered, they would be displayed here and you will be redirected back to the Get Started page once you click “ok”. Otherwise, if everything is ok click “continue” and you will be redirected to the Comparison page. The application removes any non-alphanumeric characters, with the exception of underscores and dashes, from the data.


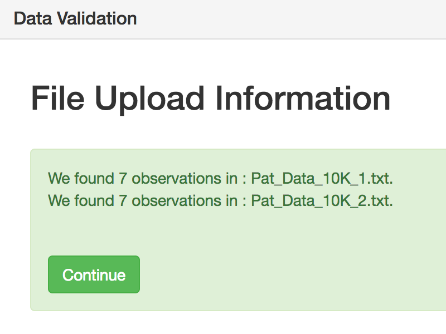


Fig. 4.3.C – File upload validation screen shot.

### 4.3.3 Comparison Page

Once data has been validated you are taken to the comparison page (Fig 4.3.D) where you can view a heatmap of all your different data files that you uploaded. There is also a pathway table near the bottom of the screen where when you select a particular pattern it will determine the pathways that go with that pattern. The selected info section to the right of the pathway table displays the pattern you selected, the file it came from, and the number of genes associated with that particular pattern.


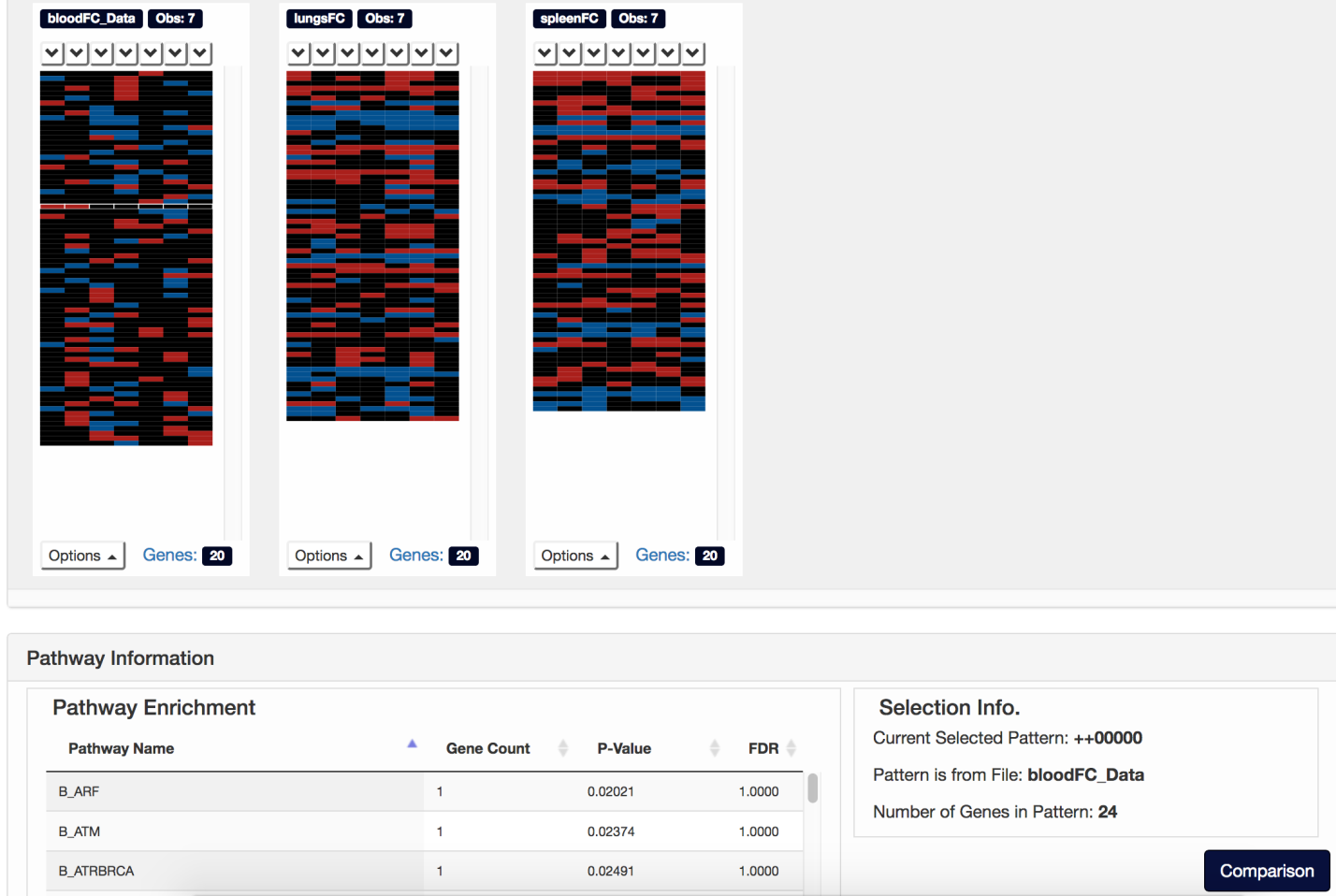


Fig. 4.3.D – Comparison page showing data file heatmaps that were uploaded with pathway data displayed for one pattern that was selected.

This comparison page allows you to look at the data file(s) to compare them. If you have multiple data files they must have the same number of observations to continue past this page.

The options button at the bottom of each heatmap allows you to sort your data files into one of three ways, either sort low to high, high to low, or back to the original input. This allows you to look for particular patterns once the data has been sorted. Once you are done looking at your data files for comparison, click on the “Comparison” button to be taken to the combined page of the application.

**Changing Gene Count per Pattern**


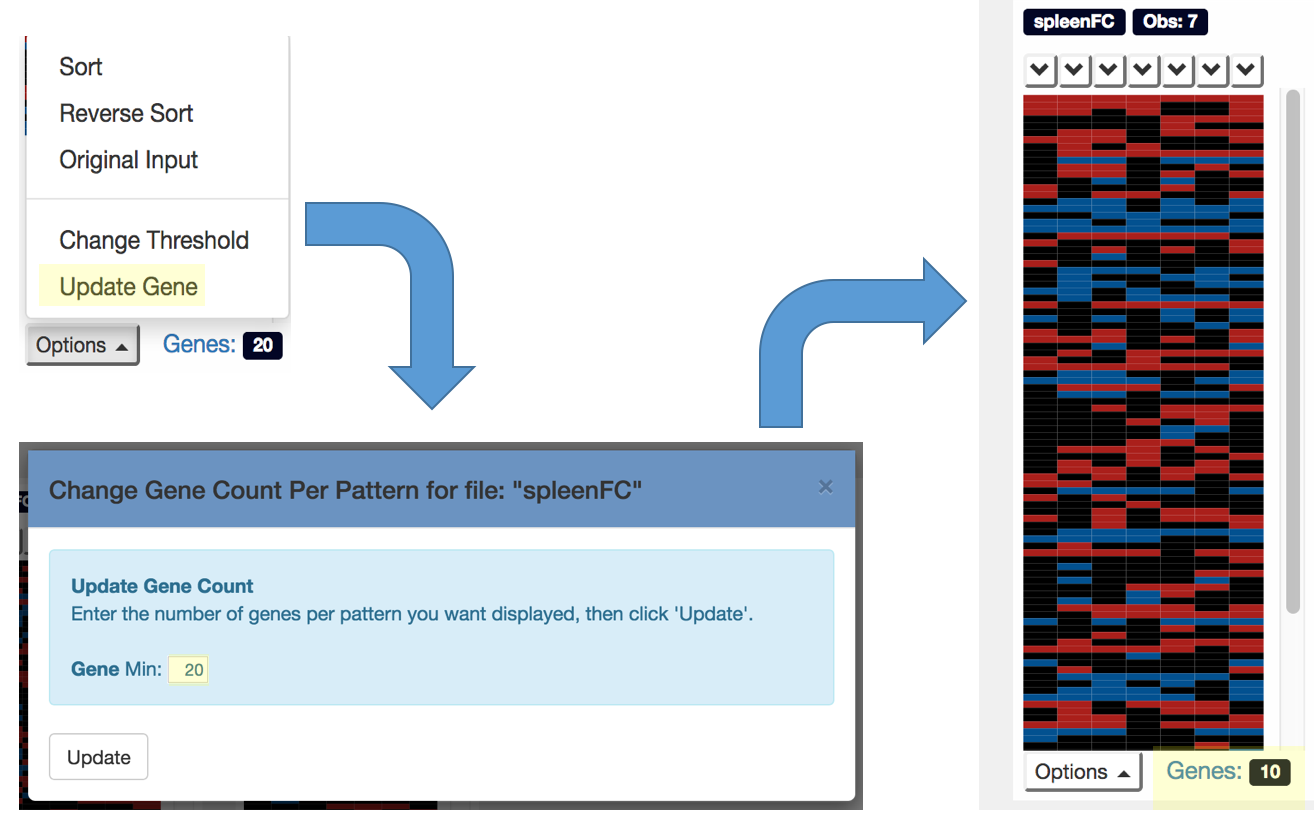


Fig. 4.3.E – Change genes per pattern flow diagram.

To change the minimum number of genes per pattern that are displayed, select the “Update Gene” option in the options dropdown menu. After selecting the “Update Gene” a change gene modal window will appear (Fig 4.3.E). After updating the gene number in the text box press the “Update” button and the modal window will close and the gene heatmap will be updated. The heatmap will now display only patterns that have the new minimum number of genes that you specified.

**Changing Pattern Threshold**


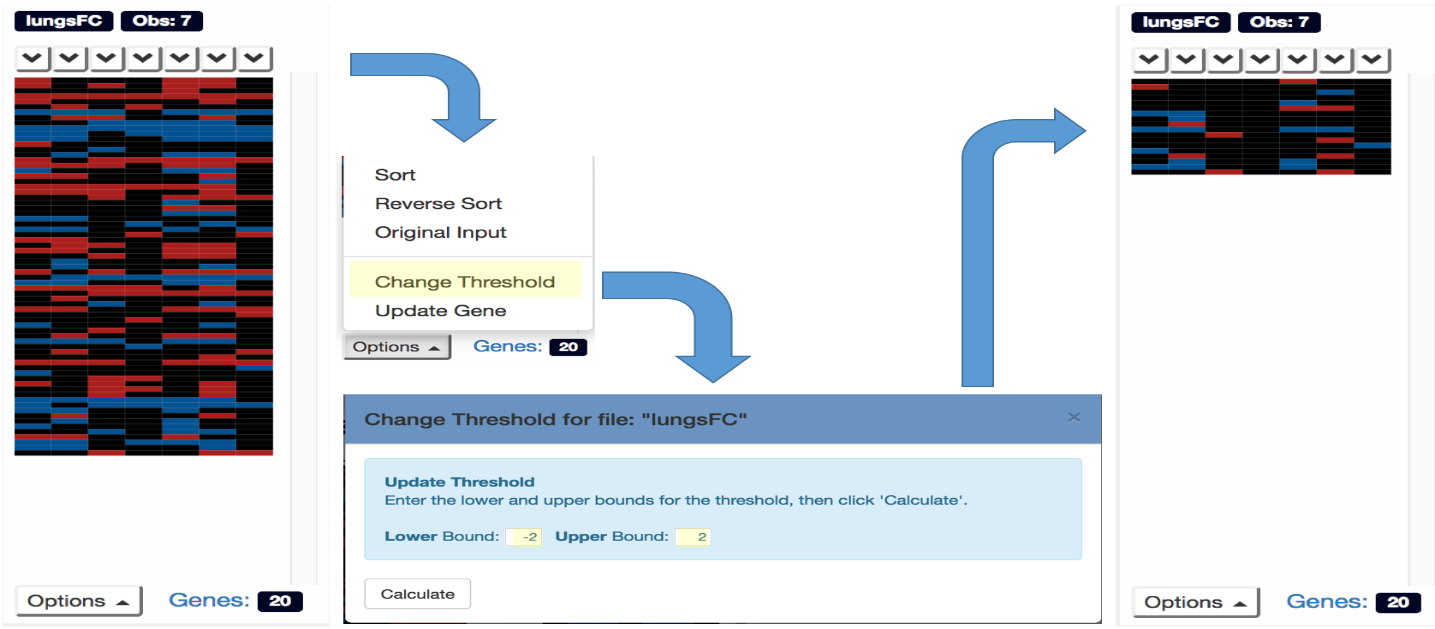


Fig. 4.3.F – Change genes per pattern flow diagram.

To change the threshold for the patterns, select the “Options” dropdown menu and select “Change Threshold” and you will be presented with a Change Threshold modal window (Fig 4.3.F). After choosing a lower and upper bound, select the “Calculate” button and the modal window will close and the gene heatmap will be updated. The heatmap will now display the new patterns with the genes clustered based on the new lower and upper bound that you specified.

### 4.3.4 Combined View

The final page of the application is called the Combined page. Here, all similar patterns from different files are combined together to display only the unique patterns among your data (Fig 4.3.H). If you uploaded one file, then this will be the unique patterns from that file. In the middle is the “Heatmap Trends” section which displays the trends associated with the pattern(s) that you select. The trends show the subtrends of the selected primary pattern(s) relative to their subsequent columns. For example, if you select a pattern which is all up “+++” there could be gene expression with values of 4, 3 and 2, meaning a downward trend, or values of 2, 3, 4 which indicates an upward trend. These are displayed in the Heatmap Trend section (Fig 4.3.H). The gene heatmap information and trend information will not be populated until a selection from the primary patterns is made, at which point it will then be filled with a heatmap for that pattern.


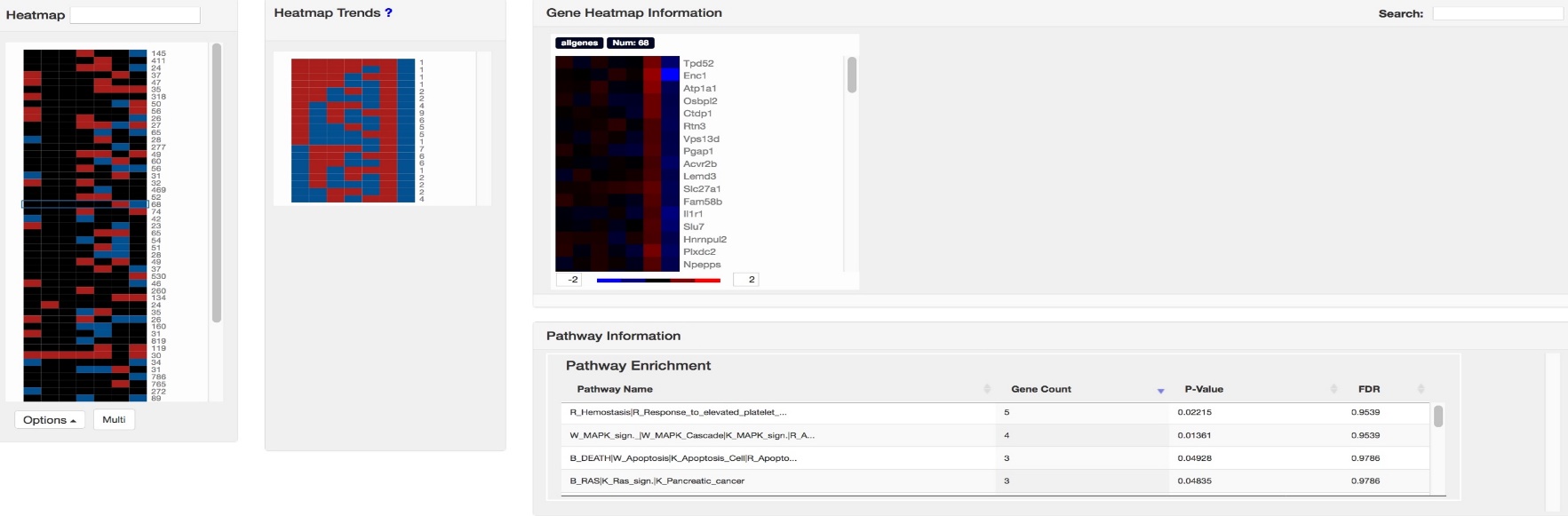


Fig. 4.3.H – Combined page showing the primary (left) heatmap, the trend heatmap (middle) and the gene heatmaps (right) from a selection.

Below the combined heatmap is a drop down “Options” button where you can sort your data in one of four ways (Fig 4.3.I).


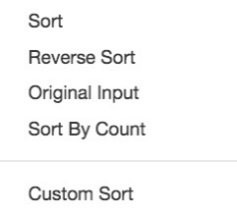

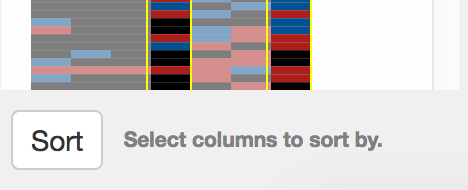


Fig. 4.3.I – Sorting choices under the Options button below the combined heatmap, and the options button changed view.

The first sorting option “Sort” arranges the patterns in a high to low fashion, “Reverse Sort” is low to high, “Original Input” sorts by the way the patterns were originally, “Sort By Count” sorts the patterns by high to low based on the number of patterns, and lastly is “Custom Sort”, which after you select it the “Options” button changes to a “Sort” button, and after you choose the columns you want to sort click on the “Sort” button. The order in which you select the columns will be the priority that they are sorted in so if you select column 4 and column 7, as in Fig 4.3.F, then it will sort the data by column 4 first then column 7 respectively. This allows you to look for particular patterns which are relevant to the scientific question you may be researching.

Once you select a pattern-of-interest, the gene heatmap area will populate with a heatmap of the gene information that makes up that pattern (Fig 4.2.B). For each data file you uploaded, there will be a corresponding gene heatmap for each file that contained the particular pattern you selected. If that file didn’t contain a particular pattern, then the gene heatmap for that file will be blank.

**Filtering Patterns & Flip Heatmap to Line Chart**

**
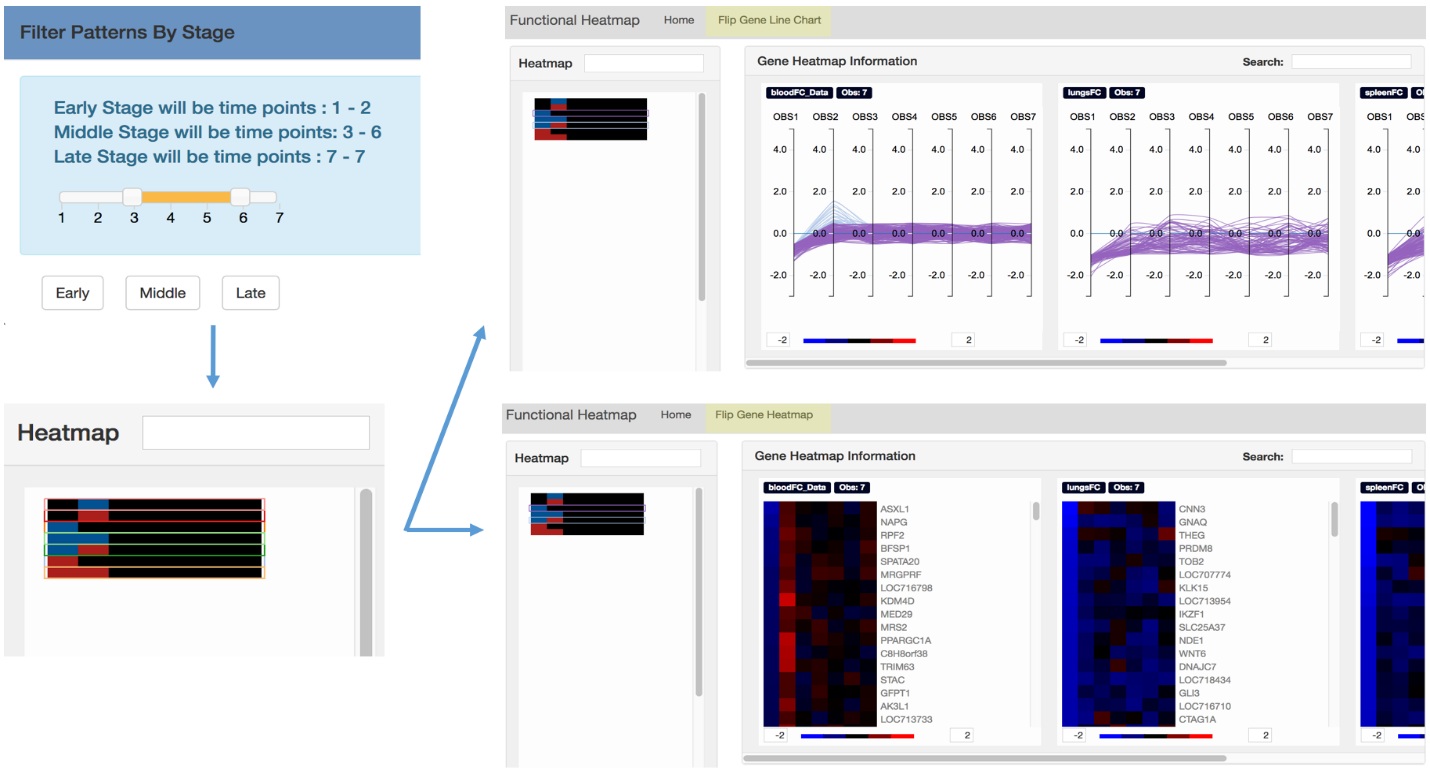
**

Fig. 4.3.F –The filtering workflow on the Combined page

When selecting the “Filter” under the “Options” menu a filter modal window displays (Fig 4.3.F) that has a slider bar with two buttons. Set the buttons to the desired time points you want and select either early, middle, or late and the patterns will be sorted and selected by those time points. You can then deselect the ones you’re not interested in and then press the “Process” button. This draws the heatmaps as before with all the patterns. You can then click on the “Flip” button in the nav-bar which will flip the gene heatmaps to parallel coordinate line charts as shown in Fig 4.3.F.


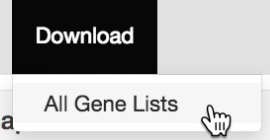


Fig. 4.3.G –The gene list download button on the Combined page

The user can download the gene list(s) of the pattern(s) they selected by clicking on the “Download” button and then selecting the “All Gene Lists” from the dropdown (Fig 4.3.G). There will be a pop-up menu asking if you want to save or open the file, so make sure you have pop-ups enabled on your browser. Then you can save or open your file, which will contain your gene information along with the pattern it came from, as well as the trend the gene is associated with.

**Pathway Selection**


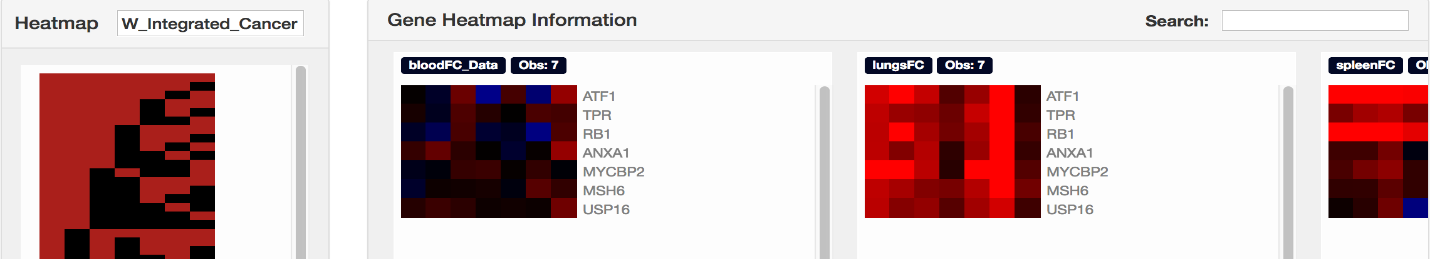


Fig. 4.3.H –Selecting pathway to get genes that match selected pathway.

You can select a pathway which will select the genes in your data that go with that pathway and draw the gene heatmaps based on your data. This feature is for when there is a particular pathway that you are interested in and want to see how the genes behaved in your data set.

The pathway information will also populate after a particular pattern is selected, which shows the pathways relevant to the genes in the selected pattern. You can narrow down the gene list to see only the genes associated with a particular pathway from the table by selecting a pathway in the table (Fig 4.2.C). This will only display in the gene heatmap(s) the genes that caused such pathway to be found in the table. Any genes which did not return a pathway are labeled “unclassified” in the table because the genes were in the list, but did not get matched up with any particular pathway.


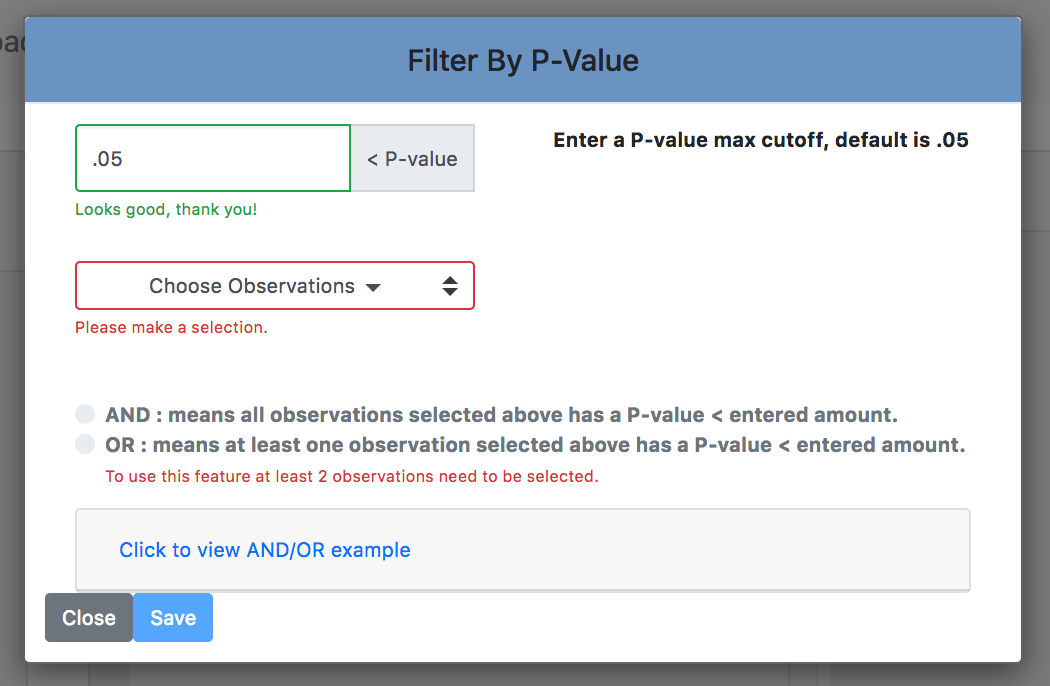

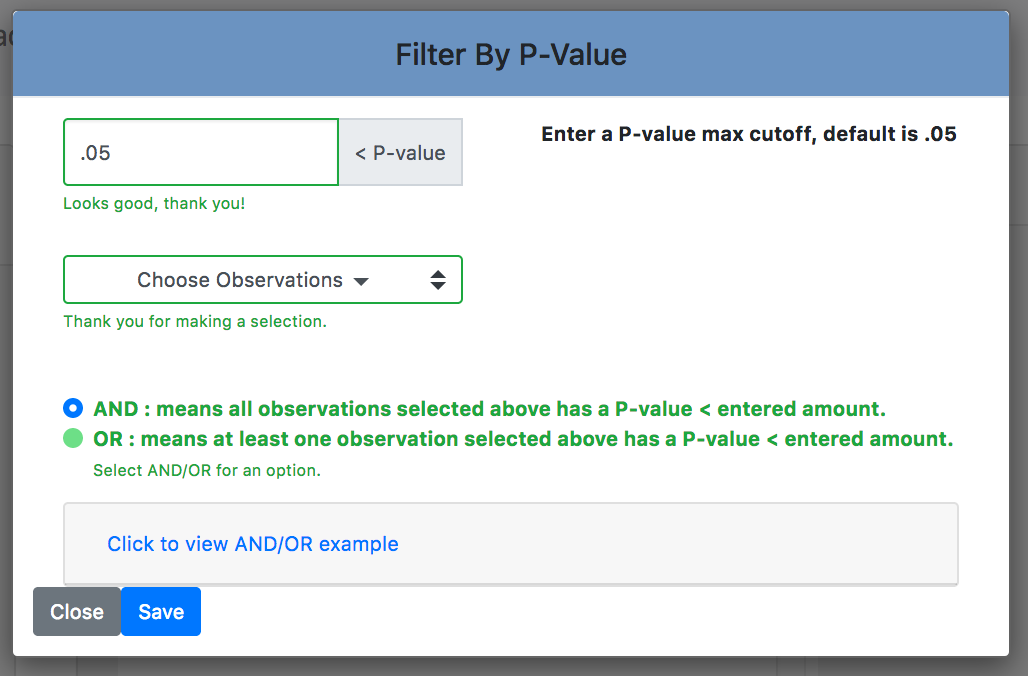

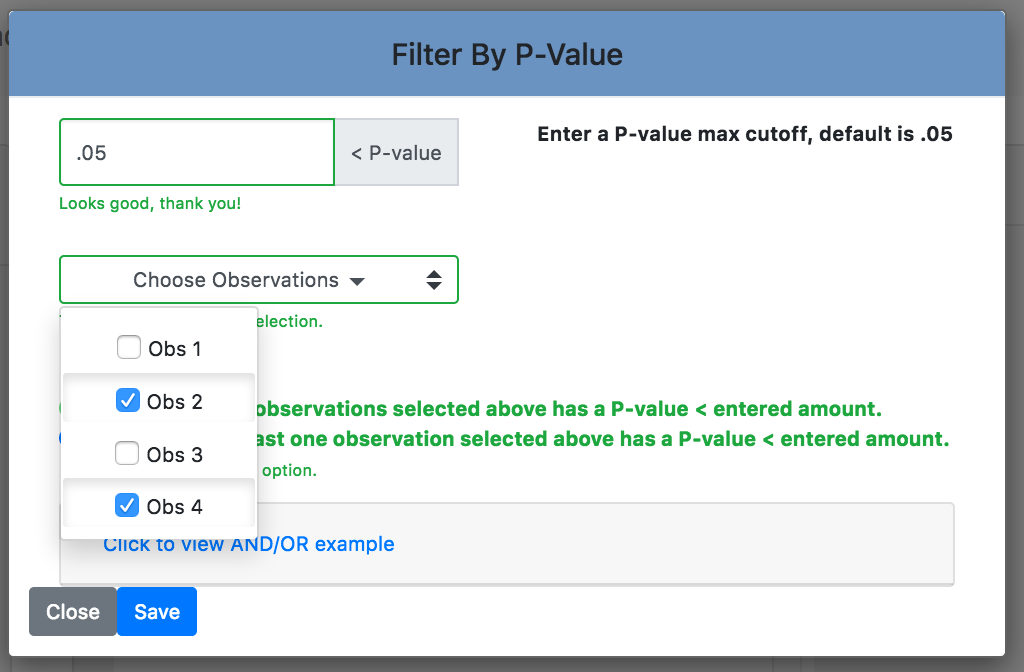


## 4.4 Contact Page

Fig. 4.3.I –P-Value filter feature.

Users can use the P-value filter feature under the Options drop down menu on the Combined page which will popup another window (Fig4.3.I) allowing the user to specify a p-value cutoff, which observation data points to apply the filter to, and whether it will be a conjunction “And” or disjunction “Or”. This allows the user to see only patterns which have genes that meet their specified statistically significant p-value cutoff point.

## 4.4 Contact Page

This page simply provides the contact information for the team and links you can click on to send us emails.

- Joshua Williams, Visualization Development & UX/UI Design

[Joshua.Williams2@NIH.gov](mailto:Joshua.Williams2@NIH.gov)

- Daniel Watson, Visualization Development & UX/UI Design

[Danie.Watson@NIH.gov](mailto:Danie.Watson@NIH.gov)

- Ruoting Yang, Algorithm & Visualization Development

Ruoting.Yang@NIH.gov
